# Singular Value Decomposition for Removal of Cardiac Interference from Trunk Electromyogram

**DOI:** 10.3390/s21020573

**Published:** 2021-01-15

**Authors:** Elisabetta Peri, Lin Xu, Christian Ciccarelli, Nele L. Vandenbussche, Hongji Xu, Xi Long, Sebastiaan Overeem, Johannes P. van Dijk, Massimo Mischi

**Affiliations:** 1 Department of Electrical Engineering, Eindhoven University of Technology, 5600 MB Eindhoven, The Netherlands; ciccarelli.1543133@studenti.uniroma1.it (C.C.); h.xu2@tue.nl (H.X.); x.long@tue.nl (X.L.); s.overeem@tue.nl (S.O.); J.P.v.Dijk@tue.nl (J.P.v.D.); m.mischi@tue.nl (M.M.); 2 School of Information Science and Technology, ShanghaiTech University, Shanghai 201210, China; xulin1@shanghaitech.edu.cn; 3 Center for Sleep Medicine, Kempenhaeghe, P.O. Box 61, 5590 AB Heeze, The Netherlands; VandenbusscheN@kempenhaeghe.nl; 4 Department of Orthodontics, University of Ulm, 89081 Ulm, Germany

**Keywords:** singular value decomposition, trunk electromyography, quantitative assessment of performance, electrocardiograph interference, respiratory monitoring

## Abstract

A new algorithm based on singular value decomposition (SVD) to remove cardiac contamination from trunk electromyography (EMG) is proposed. Its performance is compared to currently available algorithms at different signal-to-noise ratios (SNRs). The algorithm is applied on individual channels. An experimental calibration curve to adjust the number of SVD components to the SNR (0–20 dB) is proposed. A synthetic dataset is generated by the combination of electrocardiography (ECG) and EMG to establish a ground truth reference for validation. The performance is compared with state-of-the-art algorithms: gating, high-pass filtering, template subtraction (TS), and independent component analysis (ICA). Its applicability on real data is investigated in an illustrative diaphragm EMG of a patient with sleep apnea. The SVD-based algorithm outperforms existing methods in reconstructing trunk EMG. It is superior to the others in the time (relative mean squared error < 15%) and frequency (shift in mean frequency < 1 Hz) domains. Its feasibility is proven on diaphragm EMG, which shows a better agreement with the respiratory cycle (correlation coefficient = 0.81, *p*-value < 0.01) compared with TS and ICA. Its application on real data is promising to non-obtrusively estimate respiratory effort for sleep-related breathing disorders. The algorithm is not limited to the need for additional reference ECG, increasing its applicability in clinical practice.

## 1. Introduction

Trunk electromyography (EMG) is a non-invasive measure of the electrical activity of trunk muscles such as diaphragm, pectoralis, and intercostal muscles involved in respiration. The use of trunk EMG to non–obtrusively estimate the respiratory effort is promising for the diagnosis of sleep-related breathing disorders [1,2]. The application of trunk EMG to respiratory monitoring is strongly hampered due to the contamination interference of cardiac electrical activity (ECG) that distorts both the amplitude and frequency content of the signal [3,4]. The technical challenge is not trivial since the ECG amplitude overcomes the muscular electrical activity by several decibels. Typical values of the signal-to-noise ratio (SNR) on trunk EMG are around 10 dB to 20 dB, depending on the electrode position [3,5]. To monitor respiratory effort in clinical practice, obtrusive recording of esophageal pressure is considered more reliable than non-obtrusive trunk EMG and used as the gold standard [2].

In the last few decades, several algorithms have been proposed and tested to address the off-line removal of ECG interference from trunk EMG. In clinical practice, the most widespread methods are based on the gating technique (GT) and high-pass filtering (HPF). GT is based on the definition of a window around the ECG R peak that is forced to assume null values [6,7]. The length of the windows is defined to include the most power-contributing region of the ECG waveform and is typically around 0.1 s [6]. Despite its wide spread use, the GT intrinsically results in a loss of useful EMG data around each QRSpeak and is critical for high heart rates (e.g., in patients with tachycardia, preterm infants) [8]. HPF applies a filter to suppress low frequency components, considered to be mainly associated with the ECG. The EMG spectrum spans a bandwidth from 5–450 Hz, while the ECG spectrum is mostly concentrated below 25 Hz, although substantial power is distributed across most of the EMG spectrum [9]. The choice of the cut-off frequency should be carefully considered as a trade-off between EMG components to be maintained and ECG components to be eliminated. A cut-off frequency of 30 Hz is generally considered acceptable as suggested by Redfern et al. [10] and recently confirmed by our previous study [11].

A more advanced method originally proposed by Bloch in 1983 [12] is templating subtraction (TS) [7,13,14]. The algorithm aligns and averages the QRS complexes to obtain an ECG template that is subtracted from the original signal. The main assumptions of the TS method are that the ECG has quasi-periodic characteristics and that the EMG has a zero mean Gaussian distribution. Nevertheless, this last hypothesis is not always verified.

In recent years, blind source separation (BSS) methods have also been explored to discriminate signals coming from instantaneous mixing of different sources such as ECG and EMG signals [15,16,17]. A multi-channel strategy is usually adopted for BSS. This class of techniques assumes that the number of sources (physiological and artifact sources) to be detected is smaller than the number of recorded signals. The process to extract the signal of interest consists of three steps: identification of the bases that provide the best separation of the sources, identification and removal of the undesired components from the signal, and mapping the remaining components back to the original domain. A challenging aspect using BSS is the choice of the components containing the desired signal in the second step. Among the most frequently used BSS techniques, singular value decomposition (SVD) is widely used in various fields (e.g., extraction of fetal ECG from abdominal leads [18], clutter suppression in ultrasound imaging [19]). SVD decomposes the signal into a set of singular vectors associated with scaling factors called singular values (SVs), dependent on the energy of the correspondent signal. While SVs are unique, singular vectors can be determined through different algorithms, such as bidiagonalization and Jacobi methods [20]. Strictly linked to SVD, principal component analysis (PCA) is a method for dimensionality reduction traditionally based on eigendecomposition of the covariance matrix [21]. Covariance matrix computation and eigenvalue decomposition can have high computational costs [22], so SVD is often used to perform PCA [22]. Until now, SVD has not been investigated in the field of ECG canceling in trunk EMG, while two studies have proposed SVD to remove white noise from ECG [23,24]. The choice of the optimal number of SVs required to reconstruct the signal is not trivial. This number might vary for signals with different SNRs.

Independent component analysis (ICA) is a multivariate statistical approach to BSS that aims to distinguish statistically independent source signals from a set of their linear combination. ICA maximize the non-Gaussianity of the sources mainly using kurtosis, (neg)entropy, and mutual information cost functions [25]. Different from PCA and SVD, ICA’s subspaces are not necessarily orthogonal. ICA separation is accurate when the desired and undesired sources are independent. This is likely the case for additive noise, while other noise sources, such as multiplicative noise, can be interdependent with the desired signal. ICA was proposed for ECG denoising of trunk EMG under the assumption that EMG and ECG signals are independent [16,17,26,27,28,29]. In previous studies, the mixing matrix of the ICA algorithm was extracted by a synthetic dataset composed by combining the signals simultaneously recorded from multiple electrodes (from eight [27] to 16 [16,17] channels). This limits ICA-based approaches to applications with multiple sensors. The selection of the number of sources and of the component(s) related to ECG is traditionally performed by a trained professional in charge of a visual inspection of the components. Automated methods have also been proposed with promising results [16,17].

Methods based on wavelet decomposition and adaptive filtering have also been proposed to denoise trunk EMG [30,31,32]. The major challenge of the first method is the selection of the appropriate mother wavelet and the corresponding decision thresholding. The second one needs to use an appropriate reference signal for the ECG. Our recent work [11] showed that these two techniques underperform ICA-based methods and templating in denoising trunk EMG.

Despite the number of different techniques proposed in the literature to remove ECG contamination from trunk EMG, the quality of the reconstructed EMG is often not sufficient for practical applications [2,11].

The aim of the present study is to develop an SVD-based algorithm able to improve the removal of ECG interference from trunk EMG, with respect to standard methods proposed in the field (gating technique, high-pass filtering, templating subtraction, and the ICA-based method). We focused the work on methods for off-line applications and data labeling, which are commonly employed in clinical practice for the review of long recordings. As such, the approach is not optimized for real-time applications. A synthetic dataset was used to assess the performance of the algorithm with a reference signal. The algorithm does not need multiple channels to separate ECG from EMG. Multidimensional data were artificially created by segmentation of a single sensor’s signal. The novel algorithm was compared in the time and frequency domains with reference algorithms. The metrics chosen were the relative mean squared error (MSEr) and the shift in mean frequency (MF). The MSEr was used to assess the error introduced in terms of amplitude, commonly required to estimate the muscular contraction level. MF is a widely used metric to analyze the error in the frequency domain, and it is a recognized method to estimate myoelectric fatigue [33]. The algorithm was also tested on real data from the illustrative application of respiratory EMG. The respiratory pattern estimated via esophageal manometry was used as a qualitative reference for this single-patient assessment. The present work is organized as follows. Section 2 describes the dataset used and its motivation, the metrics for comparison, the novel SVD-based algorithm, and the reference algorithms. Section 3 and Section 4 report on and discuss the results, the limitations, and possible future developments. Finally, Section 5 provides the final remarks.

## 2. Materials and Methods

### 2.1. Synthetic Trunk EMG

**Figure 1:**
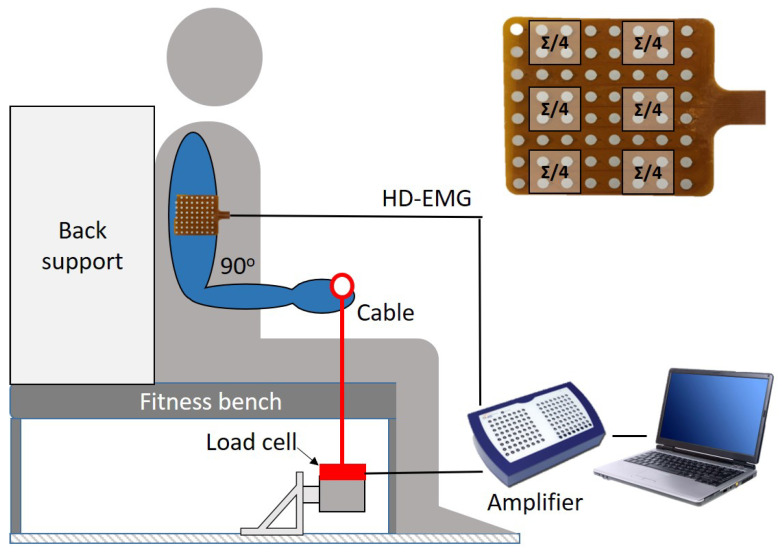
Schematic representation of the acquisition setup used to acquire the high-density (HD) EMG. Adapted from [35].

A synthetic dataset was built to simulate trunk EMG. Through this approach, we were able to obtain a reference signal to assess the performance of the algorithm. EMG data were collected on the biceps brachii of nine healthy subjects’ dominant arm by a Refa amplifier (TMS International, Enschede, The Netherlands, sampling frequency of 2048 Hz). A circular (1 cm diameter) Ag/AgCl electrode was placed on the subject’s right clavicle as the ground. A high-density (HD) EMG grid (8 × 8 channels, diameter of each electrode of 4 mm, and inter-electrode distance of 8 mm) was positioned longitudinally to the muscle fibers. Participants were seated with the back straight and the elbow joint at 90 degrees. During a 60 s isometric exercise, subjects were asked to produce a constant force equal to 50% of their maximal voluntary contraction (MVC). The acquisition protocol was composed of a 10 s rest, a 15 s 50% MVC, a 10 s rest, a 15 s 50% MVC, and a 10 s rest. The experimental setup is shown in Figure 1. Further details on the acquisition protocol were described in detail in previous works from our group [34,35]. The protocol was approved by the Ethical Committee of the Máxima Medical Center (Veldhoven, the Netherlands). To obtain a synthetic trunk EMG able to mimic the ECG overlap on the EMG signal recorded on the trunk, six EMG channels were extracted as the average of a subset of four neighbor channels from the HD grid, as shown in Figure 1. The signals were resampled at 1000 Hz to match the sampling frequency of the ECG signal.

The ECG signal was obtained by randomly selecting nine healthy datasets from the PTBDiagnostic ECG Database of Physionet [36]. Among the 15 signals included, the six precordial leads (v1 to v6) were chosen as they were measured on the chest with respect to Wilson’s central terminal. The sampling frequency of the signal was 1000 Hz.

For both the ECG and EMG signal, a second-order infinite response filter was used to remove the power line interference and a third-order Butterworth band-pass filter to remove frequency components outside the frequency band of interest (5–450 Hz for the EMG and 0.5–120 Hz for the ECG) [9,37]. The mixed signal was then obtained as the sum of the EMG and ECG re-scaled to obtain realistic SNR values (20log($\sigma_{ECG}$/$\sigma_{EMG})$) between 0 dB and 20 dB.

### 2.2. Real Data from Trunk EMG

The feasibility of the algorithm was also tested on one illustrative real recording from a patient suffering from obstructive sleep apnea (male, 69 years, body mass index of 30.4 kg/m^2^). The data were recorded at the Ethical Committee of the Máxima Medical Center (Veldhoven, the Netherlands). The participant was informed about the purpose of the study and gave his written informed consent. The protocol was approved by the Ethical Committee of Kempenhaeghe Sleep Disorder Center.

The patient underwent a full-night polysomnography within the SOMNIA project (Sleep and OSAMeasuring with Non-Invasive Applications), which collected data from patients with a wide range of sleep disorders [38]. Two monopolar Al/AgCl electrodes were positioned on the 6th intercostal space (Compumedics Grael, sampling frequency = 512 Hz). The bipolar derivation was considered to reduce the SNR. The signal was band-passed (third-order Butterworth filter with a cuff-off frequency of 5–450 Hz). In order to have a reference signal, the performance of the algorithms was compared in terms of the correlation coefficient with simultaneously recorded esophageal pressure, as the gold standard signal to monitor the respiratory cycle. The esophageal pressure was measured using a catheter located transnasally in the distal part of the esophagus (Gaeltec S7d, sampling frequency = 128 Hz).

### 2.3. Performance Metrics

To quantitatively assess the performance of the algorithms, we evaluated the error in the reconstructed signal both in the time and frequency domain analyzing two widely used EMG features, the relative mean squared error (MSEr) and the mean frequency difference (MFD).

For each channel, the MSEr was computed as:

(1) $$\mathrm{MSEr}=\frac{\sum_{n=1}^{N}{\left({\mathrm{EMG}}_{n}-{\mathrm{recEMG}}_{n}\right)}^{2}}{\sum_{n=1}^{N}{\left({\mathrm{EMG}}_{n}\right)}^{2}}\%$$

where *N* is the number of samples in the signal, ${\mathrm{EMG}}_{n}$ is the ground truth signal from biceps, and ${\mathrm{recEMG}}_{n}$ is the reconstructed EMG after the application of the algorithm. The mean value obtained for the six channels was computed for each subject.

In the frequency domain, we assessed for each channel the difference in terms of the mean frequency (MFD) induced by the algorithm. The mean frequency (MF) was computed as the first statistical moment of the EMG amplitude spectrum ($S_{n}$) obtained with the short time Fourier transform (STFT) as:

(2) $$\mathrm{MFD}\left[Hz\right]={\mathrm{MF}}_{\mathrm{EMG}}-{\mathrm{MF}}_{\mathrm{recEMG}},\;\mathrm{MF}=\frac{\sum_{n=N_{1}}^{N_{2}}f_{n}S_{n}}{\sum_{n=N_{1}}^{N_{2}}S_{n}}.$$

with *n* the STFT point, with $N_{1}$ and $N_{2}$ designed to obtain $f_{n}$ spanning from 5 to 450 Hz. The mean value obtained for the six channels was computed for each subject.

Statistical analysis was performed to verify the significance of the difference between the performance obtained with the proposed algorithm and the other algorithms. After assessing the non-normality of the distribution, a Bonferroni-corrected non-parametric Wilcoxon test for paired samples was applied on the results obtained with the SVD algorithm and with previously published algorithms (4 paired comparisons). The results are reported as the median and interquartile values.

### 2.4. SVD for ECG Denoising

The first step needed for the algorithm proposed in the present work is the detection of the QRS complexes, implemented following the algorithm proposed by Varanini and colleagues [39]. The advantage of this method with respect to a traditional peak detector is that it is more robust to a low SNR.

**Figure 2:**
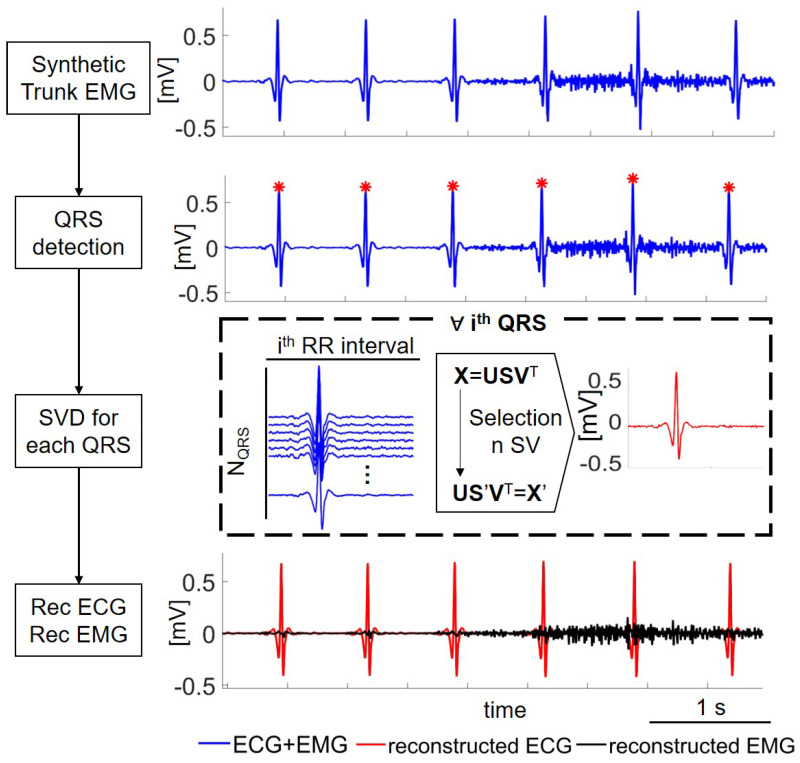
Schematic of the algorithm proposed with an illustrative synthetic trunk EMG (SNR = 10 dB). The EMG signal during the first three QRScomplexes was the minimum (rest phase), while the last three QRS were overlapped with the EMG at 50% maximal voluntary contraction (MVC). Firstly, the synthetic trunk EMG signal was processed to find the QRS complexes. For each $i^{th}$ QRS, a matrix **X** was built. Each row of **X** was composed by a single QRS centered 0.3 s before the R peak, with the length equal to the current RRinterval. Finally, the ECG component was extracted through inverse SVD considering *n* singular values, and it was subtracted from the mixed EMG.

Once the location of the R peaks is known, the algorithm applies the SVD to a matrix **X** that is updated for each *i*-th QRS complex, as shown in Figure 2. Each row of **X** is composed by a single QRS positioned 0.3 s before the R peak, with a length equal to the current R-R interval. The number of rows in **X** is equal to the number of QRS complexes considered in the decomposition. This number was optimized as described in the following section.

The SVD technique [40] allows decomposing the matrix **X** as:

(3) $$X_{rxc}=U_{rxr}S_{rxc}V_{cxc}^{T}$$

where **U** and **V** are unitary matrices and **S** is a pseudo-diagonal matrix containing the SVs as diagonal elements in descending order.

**Figure 3:**
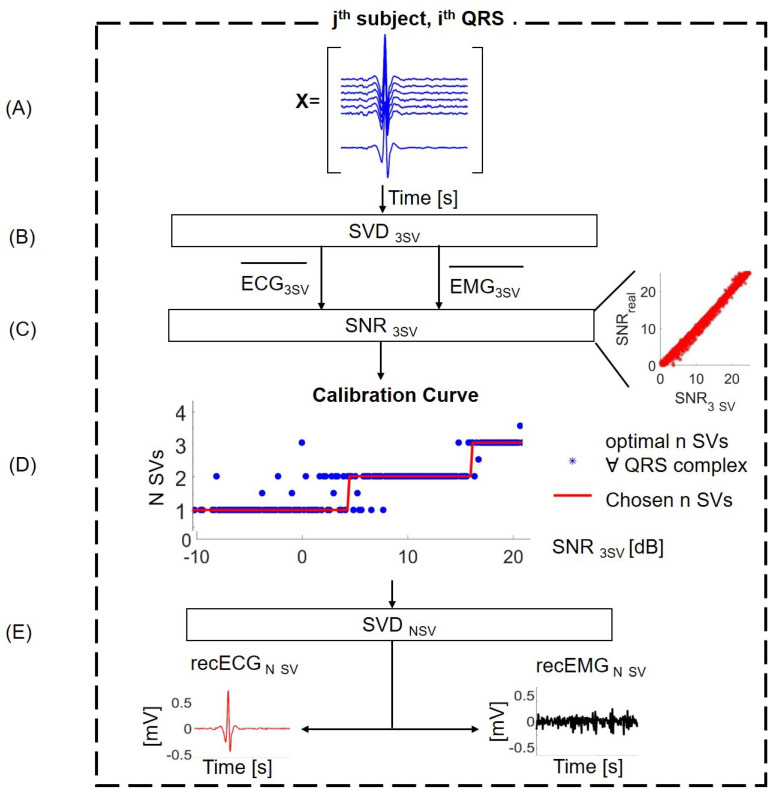
The choice of the optimal number of SVs. (**A**) Synthetic dataset with EMG + ECG signals around a single QRS complex; (**B**) application of the algorithm described in the previous section selecting three SVs to obtain an estimation of the EMG and ECG components; (**C**) SNR estimation considering ${\mathrm{ECG}}_{3SV}$ and ${\mathrm{EMG}}_{3SV}$; this approximation stands since there is a linear relationship between the real SNR and the estimated SNR with ${\mathrm{ECG}}_{3SV}$ and ${\mathrm{EMG}}_{3SV}$ shown in the figure (Panel C—on the right); (**D**) calibration curve: the choice of the optimal number of SVs on the basis of the approximation to step functions; (**E**) application of the algorithm with the appropriate number of SVs.

The selection of the optimal number of components to be used with the inverse SVD to reconstruct an ECG template is crucial to minimize the reconstruction error. Considering too few components might result in disregarding part of the ECG, while taking into account too many components would result in incorporating a part of the EMG signal in the ECG template. In the present work, we selected the number of components to be used to reconstruct the ECG template according to the SNR, following an experimental calibration curve obtained as described in a later section. The procedure followed by the algorithm for each QRS complex (dashed box of Figure 2) is better detailed in Figure 3. The estimation of the SNR was obtained by reconstructing the QRS with 3 SVs. This approximation was shown to be reliable in the range of the SNR of interest for the present work (0–20 dB), as depicted in Figure 3, Panel C. Using the calibration curve, it was possible to estimate the optimal number of SVs to apply the inverse SVD and to reconstruct an ECG template. This template is subtracted from the trunk EMG, obtaining the cleaned EMG component.

#### 2.4.1. Optimizing the Number of QRS Complexes

**Figure 4:**
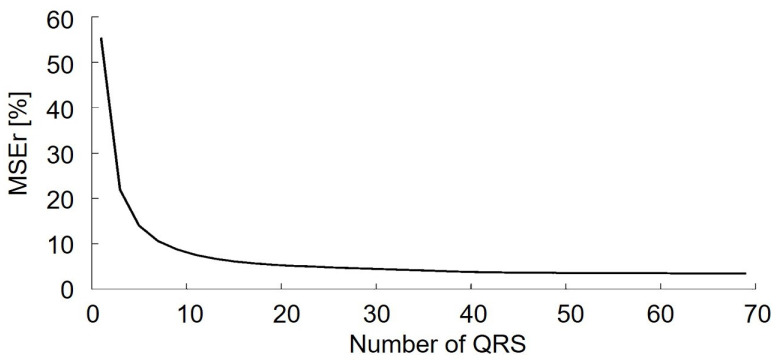
Relative mean squared error (MSEr) in the reconstruction selecting an increasing number of QRS complexes in the X matrix.

To optimize the algorithm, the optimal number of QRS complexes (number of rows in the matrix **X**) to be included in the reconstruction of a single QRS complex was investigated. A synthetic trunk EMG with SNR = 10 dB was used. The MSEr obtained considering different possible dimensions *r* of the matrix $X_{rxc}$ is shown in Figure 4.

Increasing the number of QRS complexes resulted in an exponential reduction of the MSEr up to values of MSEr = 4%. The inclusion of more than 50 QRS complexes resulted in an improvement of the performance of the algorithm in terms of an MSEr lower than the 1%. For this reason, the present work proposes to use 50 QRS complexes to apply the SVD.

#### 2.4.2. Optimizing the Number of SVs

The optimal number of components included in the inverse SVD is dependent on the SNR of the trunk EMG. In the current work, we propose to use a calibration curve to establish the best number of SVs as a function of the SNR, estimated for each QRS complex.

The calibration curve was obtained by reconstructing the QRS template with all the possible number of SVs (from 1 to 50). The SNR of the QRS complex is thus related to the number of SVs that obtained the lowest MSEr in the reconstruction, building the calibration curve depicted in Figure 3, Panel D. In order to avoid overfitting (testing the performance of the algorithm on the same dataset used to calibrate it), we assessed the performance of the algorithm with a leave-one-out approach on the calibration curve: for each subject, we tested the algorithm using a calibration curve built on the eight other subjects. Thus, for each subject, the algorithm was run with a different calibration curve.

**Figure 5:**
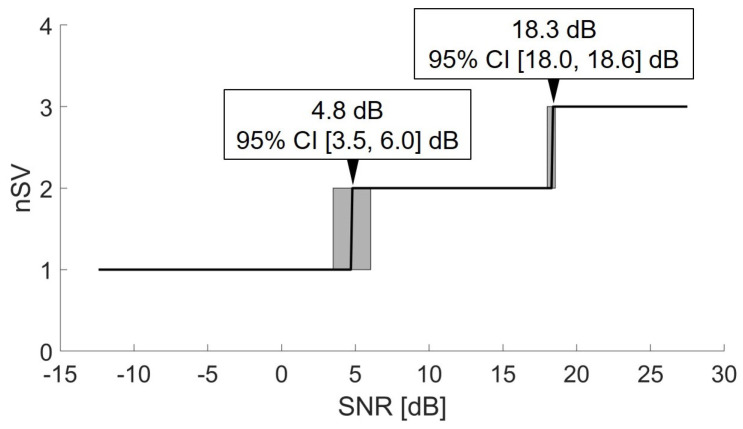
Calibration curve to establish the number of singular values to be used at each SNR level. The mean SNR and the 95% confidence interval are reported in the boxes.

The calibration curve obtained on the whole dataset is shown in Figure 5. The optimal number of SVs in the interval of the SNR spanned in the present work was from 1 to 3. The mean and 95% confidence interval (CI) are also reported in the boxes.

### 2.5. Alternative Algorithms

The performance of the SVD algorithm was compared with that of other algorithms previously proposed in literature to remove the ECG interference from EMG.

The GT was implemented as described in [7]. For each channel, the position of the R peaks was detected, and the signal around this was set to zero. The length of the QRS time interval was set as 10% of the overall RR interval duration, symmetrically distributed around the R peaks.

HPF was implemented by means of a 4th order, high-pass Butterworth filter, as described by [5]. The cut-off frequency was set at 30 Hz, as suggested by previous studies [10].

The TS was implemented considering the quasi-periodic characteristics of the ECG and assuming a zero-mean Gaussian distribution [7]. The position $t_{ij}$ of each ith R peak of the jth channel was identified, and eleven neighboring QRS complexes (5 before the QRS and 5 after the QRS) were averaged. Since the duration of the RR interval $t_{ij}^{RR}$ has an influence on the duration of the QRS, the QRS complex was averaged in the interval $\left[t_{ij}-0.25\cdot t_{ij}^{RR},t_{ij}+0.45\cdot t_{ij}^{RR}\right]$.

Finally, a fixed-point fast ICA was also implemented to distinguish EMG and ECG sources, following what was described previously [41]. The signal was decomposed as x=As, where *x* is the synthetic signal from the 6 channels, **A** is the 6×m mixing matrix, and *s* is the m×1 random vector that represents each independent source (EMG, ECG, and other noise sources). To identify the ICA components that hold information on the ECG signal, the correlation coefficients between each ICA component and an approximate ECG were computed. The approximate ECG was estimated from the synthetic signal through a 3rd-order Butterworth low-pass filter with a cutoff at 30 Hz. The two components that showed the highest correlation coefficient were selected as representative of the ECG and removed. The six-channel clean EMG was then reconstructed by multiplying the remaining components with the mixing matrix **A**.

## 3. Results

### 3.1. Performance at Different SNRs

**Figure 6:**
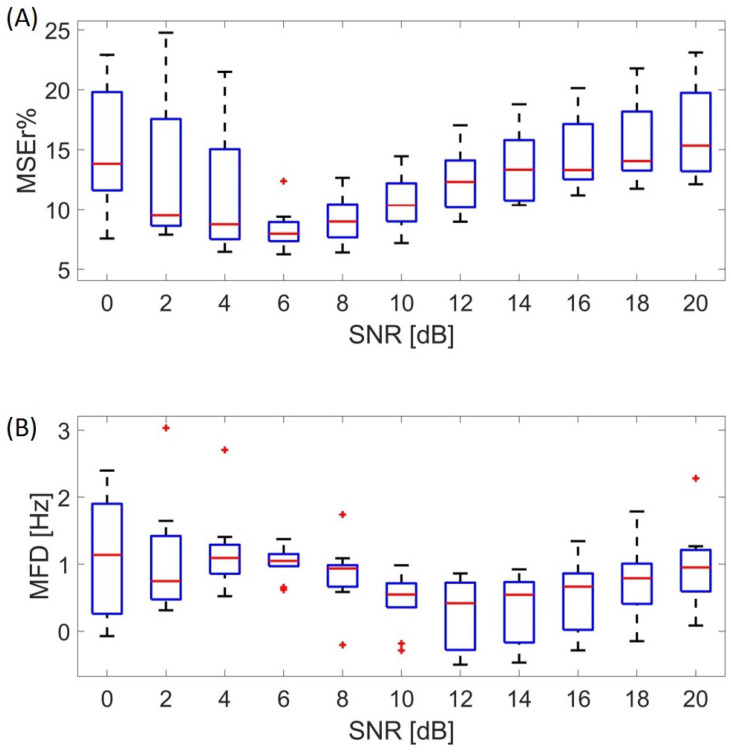
Performance of the SVD algorithm at different values of the SNR in terms of the mean squared error (**A**) and mean frequency difference (**B**). The present results were obtained on the whole signal, including the EMG from the voluntary contraction and the signal at rest. MFD, mean frequency difference.

The performance of the algorithm was tested for different values of the SNR, ranging from 0 to 20 dB. The obtained results are reported in Figure 6.

The median value of the MSEr in the time domain was below 16% for every value of the SNR (Figure 6A). The optimal performance of the algorithm was obtained with an SNR = 6 dB (MSEr = 8%). In the frequency domain, a median shift below 1.2 Hz of the mean frequency occurred for different SNRs (Figure 6B).

### 3.2. Comparison with Alternative Algorithms

**Figure 7:**
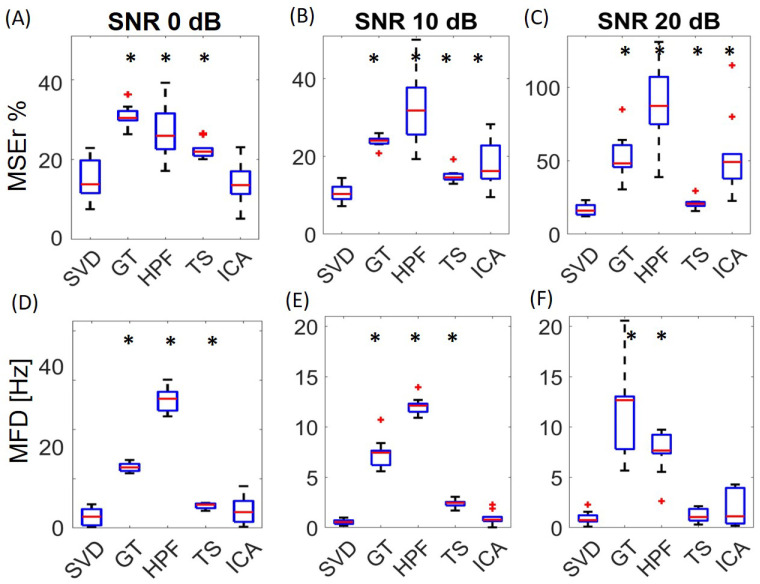
Performance of the different algorithms. SVD: singular value decomposition; GT: gating technique; HPF: high-pass filtering; TS: template subtraction; ICA: independent component analysis. The upper panels show the results in terms of the mean squared error (MSEr) with SNR of 0 dB (**A**), 10 dB (**B**), and 20 dB (**C**). The lower panels show the results in terms of the mean frequency difference (MFD) with an SNR of 0 dB (**D**), 10 dB (**E**), and 20 dB (**F**). * if *p*-value < 0.05 by the Bonferroni-corrected Wilcoxon test.

The performance of the proposed algorithm was compared in terms of the MSEr and MFD with other commonly used (GT, HPF, TS) or recently proposed (ICA) algorithms with three different levels of the SNR (0 dB, 10 dB, and 20 dB), as depicted in Figure 7. In time domain (Panels A, B, and C), the obtained results showed the superiority of SVD with respect to all the other approaches in the three conditions tested, with the exception of ICA at 0 dB, which was not significantly different from SVD.

In the frequency domain (Panels D, E, and F), the MFD obtained by SVD was always comparable or better than the other algorithms. The MFD obtained by ICA was comparable to SVD; the TS approach seemed comparable to SVD at a 20 dB SNR. The variance of the performance between subjects of SVD was lower or comparable to other algorithms.

### 3.3. Feasibility on Real Data

**Figure 8:**
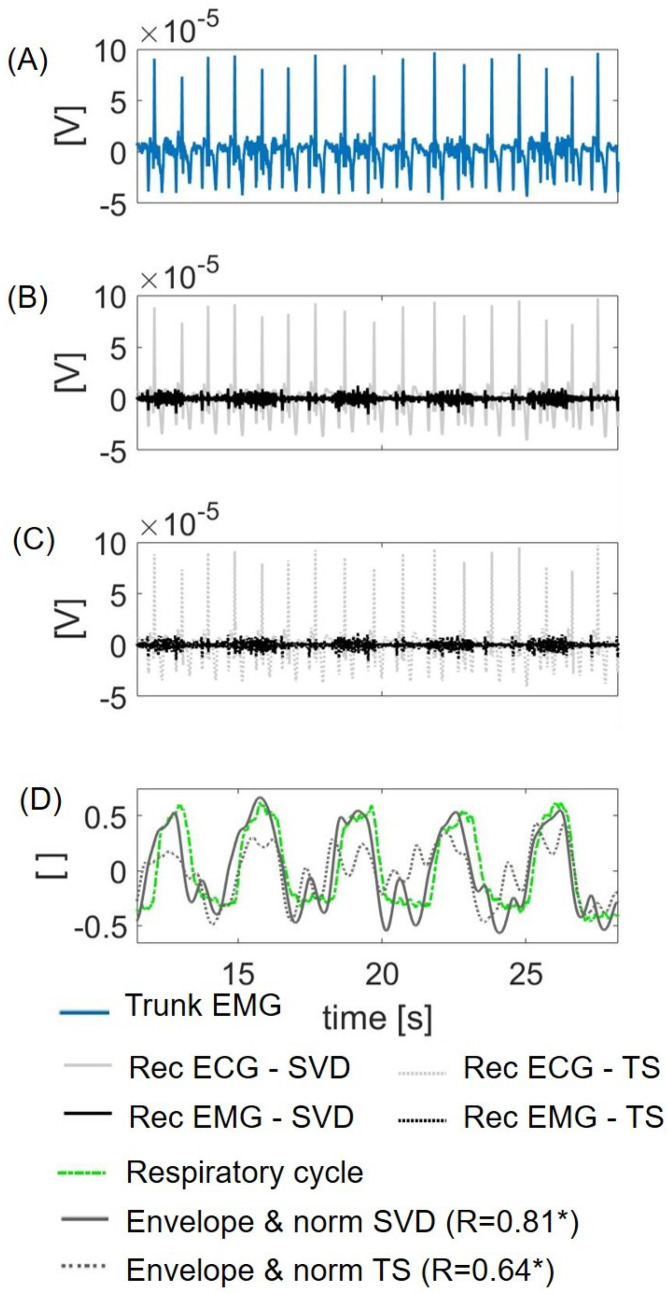
Illustrative results of the signal obtained with the algorithm applied on a real signal from trunk EMG (diaphragm). (**A**) Trunk EMG; (**B**) reconstructed ECG (Rec ECG in red) and EMG (Rec EMG in black) with the SVD-based algorithm; (**C**) reconstructed ECG (Rec ECG in dotted red) and EMG (Rec EMG in dotted black) with TS; (**D**) the envelope of the Rec EMG was computed and normalized with its maximum (gray line for the SVD-based algorithm and dotted gray line for TS). The inverted esophageal pressure is also shown (green line) as a reference of the respiratory cycle. R: correlation coefficient between EMG and esophageal pressure, * if *p*-value < 0.01.

The algorithm was tested on diaphragmatic EMG as the illustrative trunk EMG signal. The subtraction of the ECG component estimated through the SVD algorithm (Figure 8B) allowed the extraction of the diaphragmatic EMG. The envelope of the diaphragmatic EMG (Figure 8D) resulted in a signal that was highly correlated with the respiratory cycle, as estimated with the inverted esophageal pressure (correlation coefficient R = 0.81, *p*-value < 0.01). The results obtained with the TS algorithm are also shown in Panels C and D as dotted lines (correlation coefficient R = 0.64, *p*-value < 0.01). The ICA algorithm also showed a moderate correlation (R = 0.69, *p*-value < 0.01).

## 4. Discussion

An algorithm based on SVD is proposed for the removal of ECG contamination from EMG recordings. To the best of our knowledge, the current study is the first to apply SVD to (synthetic) trunk EMG to remove ECG interference. The procedure includes the identification of QRS peaks and, for each identified complex, the application of SVD to separate different components, as well as the application of inverted SVD using an optimum number of singular values. The selection of the optimal number of singular values is implemented through an experimental calibration curve that takes into account the SNR of the single QRS. The performance of the algorithm is computed both in the time and frequency domains using a synthetic dataset. The synthetic dataset is used to provide a reference signal to quantitatively assess the performance of the algorithms.

The novel algorithm was tested with different values of the SNR within a range of 0–20 dB. The results suggest that the optimal performance of the algorithm is obtained with an SNR of about 6 dB. For low values of SNR, the ECG and the EMG signal have a similar amplitude, hampering the R peaks’ detection. A misalignment of the QRS complexes in the mixing matrix results in the poorer performance of the algorithm. On the other hand, at high values of the SNR, the ECG component is predominant. Under this condition, the detection of the R peaks is trivial, but even low levels of ECG residual contamination in the reconstructed signal entail a significant amplitude error. In the frequency domain, the median frequency shift is not significantly affected by different SNRs, although a higher variability (interquartile range) of the results was shown for low and high values of the SNR.

The comparison of the SVD algorithm with other state-of-the-art algorithms highlights the superiority of the SVD approach with respect to the other investigated methods in terms of both amplitude and frequency reconstruction. GT is the simplest method, but it is strongly dependent on the dimension of the suppressing window. Short windows result in retaining part of the ECG contamination whose relevance in terms of error is proportional to the SNR. HPF introduces a significant distortion both in the time and frequency domain, resulting in the worst performance among the methods considered in the present paper. Indeed, due to the spectrum overlap between the EMG and ECG, this method seems to be unsuitable to separate the two sources.

TS produces significantly higher amplitude errors than SVD with all the tested signals, while in the frequency domain, it seems to be comparable to SVD only for an SNR = 20 dB. Previous results in the field already indicated this method as promising, identifying template subtraction as the most suitable for ECG denoising with respect to GT, HPF, wavelet transform, adaptive filtering, and ICA [11]. Nevertheless, the previous work did not consider different SNR condition of the signal in the analysis.

ICA is the only method that obtained a performance comparable to SVD in the frequency domain and the time domain (with SNR = 0 dB). ICA-based algorithms previously studied already reported a poor ability to separate the EMG and ECG, showing that about 25% of the ICA modes were ECG-contaminated [17]. The authors did not report the SNR and did not analyze the error introduced in the time or spectral content as outcome measures, making a comparison of the results difficult. In a following work investigating ICA, Willigenburg and colleagues obtained a root mean squared relative error between 10% and 30% depending on the muscular activation level [16]. The authors concluded that the performance of the algorithm was significantly improved up to a relative error between 5% and 30% by the use of a reference ECG recording. Nevertheless, in the present study, we did not consider algorithms that include the acquisition of a reference ECG signal, as this is often unfeasible in practice.

Overall, while the SVD-based algorithm outperforms the other methods in the time domain, the method is not always superior to TS and ICA in the frequency domain. Moreover, the SVD-based algorithm seems promising for all the different SNRs tested. This might be explained by the fact that the procedure was optimized to cho0se a different number of singular values for different SNR levels.

The method’s feasibility was tested on a real trunk EMG signal acquired on diaphragm muscle. Although quantitative assessment is not feasible, the envelope of the EMG reconstructed with the SVD-based algorithm shows high correlation (correlation coefficient R = 0.81) with the respiratory pattern identified through a gold standard method (see Figure 8). This result was compared with the correlation obtained through the TS and ICA, i.e., the alternative algorithms with the best performance after SVD. In this case, the correlation shown is only moderate (R < 0.7), suggesting that the use of the proposed SVD-based algorithm might provide a substantial improvement in real applications, such as overnight recording of respiratory effort to estimate sleep disorder breathing conditions, e.g., due to obstructive sleep apnea syndrome.

The present algorithm was developed for off-line and data labeling applications. Nevertheless, future studies aimed at optimizing our approach for real-time processing could be highly valuable to expand its practical application. Specifically, online reduction of noise could be addressed by singular spectrum analysis or eigen perturbation methods [42,43,44]. An optimization of the computational costs will also be needed. Possible options might be using SVD in conjunction with Kalman filtering [45].

The current study presents some limitations. Firstly, the method is based on a peak-detection algorithm to segment the signal, possibly limiting the applicability of the method to signals with either a low EMG amplitude or a high ECG amplitude. Nevertheless, within the range of possible SNRs on trunk EMG, the detector of the QRS peaks seems to be sufficiently robust. Furthermore, the algorithm was tested on a synthetic dataset. This choice was due to the need for a reference signal to quantitatively assess the performance of the algorithms. The performance of the algorithm was tested on 10 subjects only, possibly limiting the generalizability of the results. However, this sample size is comparable to or higher than those of previous studies in the field [5,14,16,28,29]. The analysis on real data, with the illustrative application of respiratory EMG, has to be considered as a feasibility analysis. It was out of the scope of the present paper to discuss thoroughly one single application. Other applications, e.g., to reconstruct the activity of back muscles, could also be considered and further investigated to foster the use of the trunk EMG in real contexts on real data. The synthetic dataset was built starting from a healthy ECG. Future studies should be carried out to assess the robustness of the algorithm on patients’ trunk EMG.

## 5. Conclusions

An novel method for the removal of the ECG interference from EMG recordings was proposed and assessed on a synthetic dataset. The performance was superior in the time domain and comparable in the frequency domain to alternative approaches currently available. When applied on real data, the algorithm seems promising in the estimation of the respiratory pattern from the diaphragm EMG. Among the strengths of the procedure proposed, no additional reference ECG is needed, increasing its applicability in clinical practice.

## Data Availability

Data are available from the authors upon request.
